# Pleasantness Only? How Sensory and Affective Attributes Describe Touch Targeting C-Tactile Fibers

**DOI:** 10.1027/1618-3169/a000492

**Published:** 2020-10-27

**Authors:** Uta Sailer, Marlene Hausmann, Ilona Croy

**Affiliations:** ^1^ Department of Behavioural Medicine, Institute of Basic Medical Sciences, Faculty of Medicine, University of Oslo, Norway; ^2^ Department of Psychotherapy and Psychosomatic Medicine, Medical Faculty, Technical University of Dresden, Germany

**Keywords:** C-tactile fibers, social touch, affective touch, gentle touch, hedonic, discriminative, tactile

## Abstract

**Abstract.** When gently stroked with velocities between 0.1 and 30 cm/s, participants typically rate velocities around 3 cm/s as most pleasant, and the ratings follow an inverted u-shape. This pleasantness curve correlates often, but not always, with the firing rate of unmyelinated C-tactile (CT) afferents, leading to the notion that CT afferents code for the hedonic or emotional aspect of gentle touch. However, there is also evidence that CT firing does not necessarily equal pleasantness, and the range of attributes that CT afferents code for is not known. Here, participants were stroked with different velocities assumed to activate CT afferents to a different extent while they rated the touch on several sensory and emotional attributes. We expected an inverted u-shaped rating curve for pleasantness and other emotional attributes, but not for sensory attributes. Inverted u-shaped rating patterns were found for the emotional attributes “pleasant” and “not burdensome,” but also for the sensory attribute “rough.” CT-directed stimulation is thus not only experienced as hedonic. The sensations arising from CTs together with all other types of mechanoreceptors might be centrally integrated into a percept that represents those aspects which are most salient for the stimulation at hand.

Recently, the role of the so-called C-tactile (CT) afferents in touch perception has gained increasing attention. These unmyelinated afferents can be found in the hairy skin of mammals and have a slow conduction velocity of about 0.9 m/s (e.g., Johansson et al., 1988; Vallbo et al., 1999; Watkins et al., 2017). They are characterized by a low response threshold (<5 mN) and a particular responsiveness to stimuli that move slowly over the surface of the skin (Bessou et al., 1971; Nordin, 1990; Vallbo et al., 1999). CTs encode tactile velocity in a nonlinear way, with responses following a negative quadratic function (inverted u-shape). In contrast, the firing rate of myelinated afferents increases with the speed of stimulation, and a positive quadratic regression model appears to provide the best fit (Ackerley, Backlund Wasling, et al., 2014; Löken et al., 2009). Just as the firing rate of CTs, ratings of subjective pleasantness are maximal for velocities between 1 and 10 cm/s and follow an inverted u-shaped pattern (Ackerley, Backlund Wasling, et al., 2014; Löken et al., 2009). This inverted u-shaped pattern of pleasantness ratings for stroking at different velocities was first reported by Essick et al. (1999) where different fabrics were moved across the participants’ skin. Many studies have since observed comparable quadratic relationships between stroking speed and mean pleasantness ratings (e.g., Ackerley, Backlund Wasling, et al., 2014; Ackerley, Carlsson, et al., 2014; Croy, Geide, et al., 2016; Essick et al., 2010; Gentsch et al., 2015; Hielscher & Mahar, 2017; Jönsson et al., 2015; Kass-Iliyya et al., 2017; Krahé et al., 2018; Löken et al., 2011; Sailer & Ackerley, 2019; Sehlstedt et al., 2016; Triscoli et al., 2013).

Since the impulse rate of CT fibers and pleasantness ratings follow this similar pattern, it has been suggested that CTs convey positive affective touch (McGlone et al., 2014; Morrison et al., 2010). CT fibers also fire maximally to slow stroking performed at skin temperature, thereby supporting the view that they are specifically tuned to respond to human caress (Ackerley, Backlund Wasling, et al., 2014). Indeed, humans spontaneously use velocities in the CT optimal range when they stroke their partner or children, but not when they stroke an artificial arm (Croy, Luong, et al., 2016).

However, in the two microneurography studies where CT firing data and pleasantness ratings were related to each other (Ackerley, Backlund Wasling, et al., 2014; Löken et al., 2009), these data were collected in separate sessions and from different participants. Thus, it is actually not known yet how pleasantness ratings and CT firing correspond to each other in the same individual. This would also be important to know because the inverted u-shape of the pleasantness curve only holds on the group level, not on the level of the single participant (Croy et al., 2020). Even on the group level, pleasantness ratings do not always correlate well with the CT impulse rate, for example, when using different temperatures (Ackerley, Backlund Wasling, et al., 2014). To sum up, the link between CTs and pleasantness is hypothesized, yet it is tenuous.

Apart from CTs, it is likely that pleasantness in touch is signaled by all types of mechanoreceptors and that the perception of pleasantness is constructed on a central level, not at the periphery. In healthy humans, any type of stroking will always activate the Aβ system along with the CT system, and the Aβ system is also capable of registering all the aspects of the touch provided. In line with this, slow stroking of the palm, where no CTs are present, is most often perceived as equally pleasant (or similarly preferable compared to other velocities) as on the hairy skin, where CTs are abundant (e.g., Ackerley, Carlsson, et al., 2014; Kaiser et al., 2016; Luong et al., 2017; Löken et al., 2011; Pawling et al., 2017; Perini et al., 2015). Also, the selective stimulation of CT afferents in a patient lacking large myelinated afferents induced only a vague sensation of pleasantness (Olausson et al., 2002). Moreover, the assumption that emotional and discriminative aspects of touch are transmitted in anatomically discrete second-order pathways has been recently questioned by findings which show that disruption of the spinothalamic tract did not change ratings of touch pleasantness (Marshall et al., 2019). It seems therefore likely that the perception of pleasant touch depends on the integration of input from both myelinated and unmyelinated afferents (see also Vallbo et al., 2016).

For these reasons, we need to understand better if and how CT activity is related to touch attributes other than pleasantness. There are a few studies which collected ratings to touch at different velocities on dimensions other than pleasantness, although without the collection of comparable microneurography data. In these studies, brushing with 3 cm/s is assumed to activate CTs more than brushing at velocities below 1 cm/s or higher than 10 cm/s. When participants were asked to rate touch provided at different velocities on a visual analog scale (VAS) with the endpoints “not erotic at all–extremely erotic” (Jönsson et al., 2015), the ratings followed a similar pattern than pleasantness ratings did. In a different study, touch at different velocities was rated according to the softness of the touch provider’s versus one’s own skin (Gentsch et al., 2015). Also in this case, the resulting patterns were similar to the typically observed rating curves for the aspect of pleasantness, namely an inverted u-shape.

As the relationship between stroking speed and ratings of eroticism and skin softness follows a similar quadratic pattern (Gentsch et al., 2015; Jönsson et al., 2015), with a maximum at speeds that are optimal to activate CT fibers, it has been suggested that CTs may also code for the erotic or softness aspect of skin-to-skin touch. It thereby seems that CT-targeted stimulation is not perceived as a specifically “pleasant” sensation. Instead, a broader variety of touch descriptors may be suited to describe the arising perception. This is also indicated by studies on the role of affective touch in communicating emotions. Instead of on their own emotions during the reception of touch, participants in these studies focused on the emotion that the touch provider may have intended to communicate. Like that, several rather different attributes than just pleasantness became relevant for describing the touch. Stroking touch compared to other types of touch was often interpreted as conveying “love” and “sympathy” (Hertenstein et al., 2006, 2009). When participants were asked to convey a “calming” emotion via touch, slow stroking was the preferred type of touch chosen and was also correctly identified as “calming” by the touch receivers (Hauser et al., 2019). Comparing CT-targeted stroking to fast stroking, participants interpreted the CT-targeted velocity as mainly conveying “arousal” and “support,” and the fast velocity as mainly conveying “warning,” but also “fear” and “joy” (Kirsch et al., 2018). Thus, stroking targeted to activate CT fibers may give rise to perceptions and emotions that can be described other than just as “pleasant.”

Further dimensions on which touch perception can be described are reported in studies on tactile textures of fabrics and surfaces. This has long been of interest. Based on observational studies, Katz (1925, 1989) identified general characteristics (Modifikationen), i.e., qualities on which any surface can be rated (e.g., soft–hard or rough–smooth), and which do not refer to a certain material. He contrasted these to specific characteristics (Spezifikationen), i.e., identifying characteristics that tell us with which particular material or fabric we are dealing (e.g., wood, leather). Following up on this distinction, Yoshida (1968) had participants rate object surfaces (e.g., glass, paper, stone) and fabrics (e.g., silk, wool, and cotton) on 20 different dimensions and concluded that the most important general descriptive dimensions were heaviness, coldness, wetness, smoothness, and hardness. Also, in later studies, the dimensions smooth–rough, hard–soft, and warm–cool appear to have been central and were completed or modified to contain other attributes such as slippery–sticky, flat–bumpy (Hollins et al., 1993), moldable–springy (Hollins et al., 2000), soft–harsh and thin–thick (Picard et al., 2003), sticky–not sticky, unpleasant–pleasant (Guest et al., 2009), and moist/dry (for a review of studies on these dimensions, see also Okamoto et al., 2013).

Distinguishing between sensory and emotional aspects of touch in the so-called touch perception task (TPT), separate factor analyses were performed on 26 sensory and 14 emotional attributes of tactile stimuli (Guest et al., 2011). The sensory attributes could be grouped into four factors (roughness, slip, pile, and firmness), and the emotional attributes into two factors (comfort and arousal). The TPT was also used to distinguish touch performed at CT-targeted velocity on two locations, the forearm versus palm (McGlone et al., 2012), as the forearm contains CT fibers and the palm does not. Brushing on the forearm was rated as more “comfortable,” less “hairy,” and less “fluffy.” Moreover, there was a tendency toward a greater use of emotional descriptors (calming, soothing, relaxing, comfortable, enjoyable, and desirable) for the forearm than for the palm (McGlone et al., 2012). This location-specific description of touch perception suggests that those attributes are well suited to describe the perception conveyed by CT fibers.

Nevertheless, it is to date not known how ratings to those attributes correspond to CT fiber activity and how touch performed at different speeds is evaluated along a broader range of sensory and emotional attributes. We hypothesized that CT-targeted stroking gives specific rise to sensations of pleasantness and other positive emotional attributes. To test this hypothesis, participants were stroked at velocities that are assumed to activate CT fibers to a different extent and rated the touch on seven different attributes. We expected that ratings for pleasantness and other positive emotional attributes would follow an inverted quadratic shape, whereas ratings of sensory attributes would not follow a quadratic shape.

## Methods

### Participants

Altogether 44 participants took part in the experiment. Exclusion criteria were skin diseases on the arm that may interfere with touch perception, neurological disorders, and depression. One participant was excluded due to a score of 17 (range 0–27) in the depression scale of the Patient Health Questionnaire (PHQ-D; Löwe et al., 2002), which indicates clinical levels of depressive symptoms. Depression served as an exclusion criterion because anhedonia, a reduced ability to experience pleasure, is characteristic for depression, and participants with subclinical higher depression have a more negative attitude to social touch (Triscoli et al., 2019). Anxiety was screened for because various forms of anxiety may modulate the response to CT-targeted touch (e.g., Krahé et al., 2016; Liljencrantz et al., 2017). None of the participants showed clinically relevant symptoms of anxiety.

The remaining 43 participants consisted of 25 women (*M* = 24.6; range 21–51) and 17 men (*M* = 26.4; range 18–35), and one person who did not want to provide that information (aged 23).

Sample size calculation with GPower (Faul et al., 2007) recommended 42 participants for a MANOVA with repeated measures within-subjects, a medium effect size of *F* = 0.25, a power of 0.8, *p*-level of .05, and a correlation between repeated measures of *r* = 0.2.

### Procedure

Upon arrival, written informed consent was obtained from each participant.

Participants were asked to fill in a questionnaire on mental health, the short form of the German Prime MD PHQ-D (Löwe et al., 2002). The PHQ-D is a 15-item instrument for the short screening of depressive symptoms, anxiety, and psychosocial functioning. The PHQ-D is validated and has very good criterion validity, particularly for the assessment of depression, with a sensitivity of 95% and specificity of 86% (Gräfe et al., 2004). The reliability of the depression scale is reported as high with Cronbach’s α = 0.88 (Gräfe et al., 2004). The participants also filled in a German version (Freitag, n.d.), validated as short version in Freitag et al. (2007), of the Autism-spectrum-Quotient (Baron-Cohen et al., 2001; data not presented here). Furthermore, participants answered questions inquiring about skin diseases and neurological disorders.

The participants were seated comfortably with their left arm resting on a pillow that was attached to a table. The left arm was chosen for stroking so that the right hand could be used to give the ratings.

The experiment consisted of seven blocks, one per attribute. In each block, 5 different velocities (0.3, 1, 3, 10, 30 cm/s) were presented 3 times and rated on one sensory or emotional attribute. The order of velocities was randomized within a block. The order of blocks was also randomized across participants. Seven blocks resulted as there were seven different attributes. After block 4, participants were given the opportunity to take an optional break of up to 10 minutes.

In each block, a different sensory or emotional attribute of the sensation was rated. The attributes used were exciting–not exciting (in German “aufregend–nicht aufregend”), burdensome–not burdensome (belastend–nicht belastend), smooth–rough (glatt–rau), hard–soft (hart–weich), cold–warm (kalt–warm), weak–intense (schwach–intensiv), and unpleasant–pleasant (unangenehm–angenehm). The word named to the right in the above description was always presented on the right end of a VAS and was coded with 10.

Five of the seven attributes were based on the TPT (Guest et al., 2011), namely the three sensory attributes: “smooth–rough” loading high on the factor roughness, “hard–soft” loading high on firmness, “cold” loading high on slip, and “warm” not loading on any factor. Emotional attributes were also selected according to the TPT: “exciting–not exciting” loading high on the factor “arousal,” and “burdensome–not burdensome” which we assumed to be related to the factor “comfort.” In addition, two commonly used attributes in studies on CT-targeted touch were used: the sensory attribute “not weak–intense” and the emotional attribute “unpleasant–pleasant” (e.g., Case, Ceko, et al., 2016; Case, Laubacher, et al., 2016; Croy et al., 2014; Ellingsen et al., 2014; Jönsson et al., 2015; Krahé et al., 2016; Mayo et al., 2018; McGlone et al., 2012; Ree et al., 2019; Rosenberger et al., 2018; Sailer & Ackerley, 2019; Sehlstedt et al., 2016; Triscoli et al., 2013; van Hooijdonk et al., 2019).

A soft goat hair brush of 23 mm width was used to stroke across a distance of 7 cm. Stroking was performed in a proximal–distal direction on the dorsal side of the left forearm. The brush was attached to a machine (“Rotary Tactile Stimulator,” Dancer Design, St. Helens, UK) controlled by LabVIEW software (National Instruments, TX, USA). The participants did not wear headphones and could therefore hear the sound of the machine. A calibrated force of 0.4 N was used for stroking. After each brush stroke, the participants were requested to rate the sensation using a mouse on a VAS ranging from 0 to 10 that was presented on a touch pad.

The data underlying this publication are not shared openly because we lack consent to open publishing from the participants. However, the data are available from the authors on request.

### Data Analysis

Six participants had incomplete datasets where the ratings of all velocities for one attribute were missing due to technical errors. This equals 2.0% of the data, and 4,530 ratings were left for the analysis in total. SPSS version 26 (IBM Corp., Chicago, IL, USA) was used to calculate the MANOVA, and MATLAB and Statistics Toolbox Release 2017b (The MathWorks, Inc., Natick, Massachusetts, USA) were used to calculate the fits.

For each participant, the three ratings per velocity and attribute were averaged. The mean rating values were submitted to a MANOVA. The ratings for each attribute were used as dependent variables (seven dependent variables: exciting–not exciting, burdensome–not burdensome, smooth–rough, hard–soft, cold–warm, weak–intense, unpleasant–pleasant), and velocity was used as a within-subject factor (five levels: 0.3, 1, 3, 10, 30 cm/s). In case of nonsphericity, the degrees of freedom were corrected with the Huynh–Feldt method. To determine the form of the mean rating curves, within-subjects contrasts were calculated. Effect sizes are reported as partial eta square (η_p_^2^).

To investigate the curve patterns separately for each participant, linear and quadratic fits together with their respective Akaike information criterion (AIC) were calculated for the single trial ratings with velocity as an independent nominal variable. Next, the AIC for the quadratic model was subtracted from the AIC for the linear model. This difference value, the delta AIC, served to determine how much better one model was respective to the other one (e.g., Symonds & Moussalli, 2011). According to the criteria proposed by Burnham & Anderson (2002), a resulting delta AIC of >4 was interpreted as no support for the linear model. Otherwise, the linear model could not be rejected or would be strongly supported if delta AIC was <2.

If delta AIC was >4, we based the further evaluation on the results of the quadratic fit, otherwise on that of the linear fit. In all cases when delta AIC was >4, the quadratic coefficient differed significantly from zero (*t*-test on the coefficient of curvature in the quadratic fit: *p* < .05). Therefore, we classified those cases as clearly curved responses. A certain fraction of these cases also showed a linear coefficient of the quadratic fit that differed significantly from zero. These cases were responses showing both linear and quadratic components in the response.

All cases with delta AIC <4 were further divided depending on the significance of the slopes of the linear fit. Cases with such a significance (*t*-test on the slope of the linear fit: *p* < .05) were classified as showing a pure linear trend, and those without were classified as cases without any clear dependence on the stimulus velocity.

To explore how the ratings of different attributes are related to each other, Pearson’s correlations were calculated between the mean ratings per velocity (average of three trials per participant) for each attribute.

## Results

**Figure 1:**
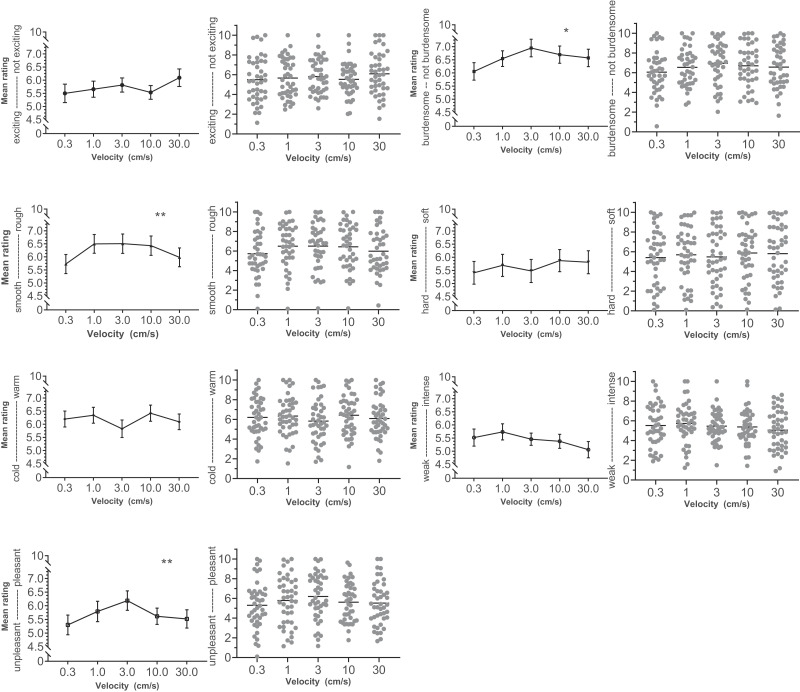
Mean with standard error for ratings of brushing velocities on different dimensions. *Note*. To the right of each curve, the means of the three individual ratings per velocity are displayed (with mean as horizontal line). A significantly inverted quadratic pattern was found for the attributes unpleasant–pleasant; burdensome–not burdensome, and smooth–rough. *p < .05; **p < .001. For each label, ratings of 43 participants were included, except for exciting–not exciting (*N* = 42), burdensome–not burdensome (*N* = 41), and smooth–rough (*N* = 40).

Depending on the respective attribute, the stroking velocities were evaluated differently. As shown in Figure 1, medium CT optimal velocities were rated higher on the attributes “unpleasant–pleasant,” “burdensome–not burdensome,” and “smooth–rough” – thus, more pleasant, less burdensome, and rougher – than very slow or very fast velocities. Visual inspection shows no such pattern of higher ratings at CT optimal velocities for the emotional attributes “exciting–not exciting,” or for the sensory attributes “hard–soft,” “cold–warm,” and “weak–intense.”

Two attributes showed significantly different ratings depending on stroking velocity: smooth–rough, *F*(3.434, 123.625) = 3.324, *p* = .017, η_p_^2^ = 0.085, and unpleasant–pleasant, *F*(4, 144) = 2.658, *p* = .035, η_p_^2^ = 0.069. Thus, for some velocities, the ratings of pleasantness and roughness were higher than for other velocities. Contrasts showed that the differences in the ratings across velocities could be approximated with a quadratic fit, but not with a linear fit. The quadratic fit for “smooth–rough” was significant at *F*(1, 36) = 9.007, *p* = .005, η_p_^2^ = 0.200, and it was significant for “unpleasant–pleasant” at *F*(1, 36) = 9.226, *p* = .004, η_p_^2^ = 0.204. The ratings for “burdensome–not burdensome” also followed a quadratic pattern, *F*(1, 36) = 4.864, *p* = .034, η_p_^2^ = 0.119, but the pattern’s curvature was not strong enough for the mean ratings to differ significantly from each other, *F*(3.504, 126.154) = 2.264, *p* = .074, η_p_^2^ = 0.059.

The results on the individual level are less clear and show that all combinations of positive, negative, linear, and quadratic rating patterns occurred. Nevertheless, it appears as if the number of participants with ratings that followed a negative quadratic fit was higher for the attribute ratings that also followed a quadratic fit in the group analysis, namely “smooth–rough,” “unpleasant–pleasant,” and “burdensome–not burdensome” (Table 1). The attribute with the highest number of positive quadratic relationships was “weak–intense.”

**Table 1:** Number of participants whose ratings followed a linear or quadratic fit or both

Attribute	Neg. quadr. + neg. lin.	Neg. quadr.+ pos. lin.	Pos. quadr. + neg. lin.	Pos. quadr. + pos. lin.	Neg. quadr.	Pos. quadr.	Neg. lin.	Pos. lin.	Percentage
Exciting–not exciting	0	2	0	1	0	0	2	1	14.3
Burdensome–not burdensome	0	1	0	0	3	1	1	2	19.5
Smooth–rough	0	1	1	1	4	1	2	1	27.5
Hard–soft	0	2	1	0	0	0	4	3	23.3
Cold–warm	0	0	0	0	0	0	1	2	7.0
Weak–intense	0	2	4	0	1	1	7	5	46.5
Unpleasant–pleasant	0	0	0	0	2	0	3	5	23.3
*Note*. Pos. = positive; neg. = negative; lin. = linear; quad. = quadratic. Numbers are listed separately for each attribute. The column “percentage” shows the percentage of participants whose ratings showed any of these fits. For each label, the ratings of 43 participants were included, except for exciting–not exciting (*N* = 42), burdensome–not burdensome (*N* = 41), and smooth–rough (*N* = 40).									

**Figure 2:**
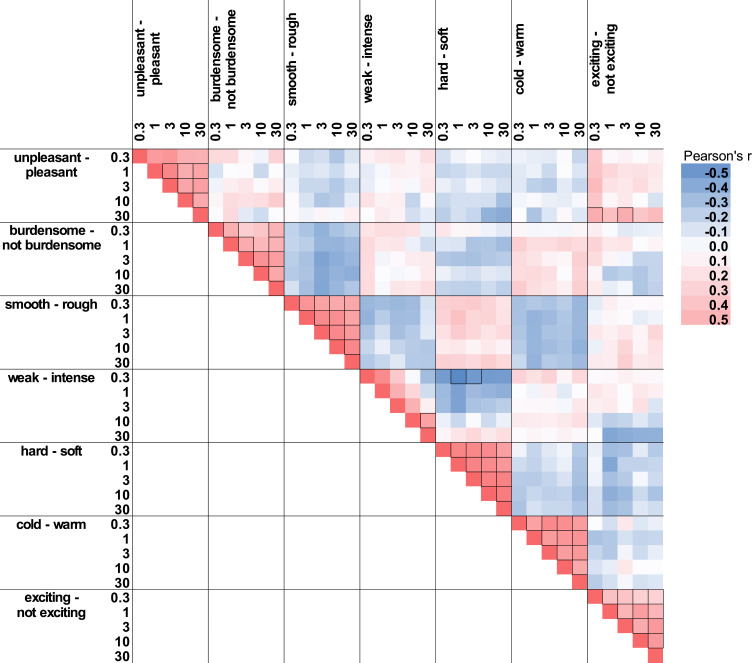
Correlation between ratings of different attributes. The more intense the color, the larger Pearson’s correlation coefficient. Negative correlations are in blue, and positive correlations in red. Significant correlations (*p*_Bonf_ < 0.0015) are marked with a frame.

Figure 2 shows that the correlations of different velocities for ratings on one attribute were higher than for the same velocity across different attributes. For example, high ratings of “pleasantness” on one velocity are related to high “pleasantness” ratings of the other velocities as well.

Looking at the different attributes, it can be seen that ratings for “pleasantness” and “burdensomeness” are less related to each other than “burdensomeness” and “roughness.” As there were few correlations which survived multiple comparison corrections (*p*_Bonf_ < 0.0015; see Figure 2), the correlation matrix should only be interpreted descriptively.

## Discussion

As in many previous studies (Ackerley, Backlund Wasling, et al., 2014; Ackerley, Carlsson, et al., 2014; Bendas et al., 2017; Croy et al., 2020; Croy, Geide, et al., 2016; Hielscher & Mahar, 2016; Jönsson et al., 2015; Luong et al., 2017; Löken et al., 2009, 2011; Morrison et al., 2011; Sailer & Ackerley, 2019; Sehlstedt et al., 2016), the relationship between velocity and pleasantness ratings followed a negative quadratic function – on a group level. This pattern of velocity-dependent ratings was, however, not limited to the attribute “unpleasant–pleasant” but was also observed for “burdensome–not burdensome” and “smooth–rough.” At the same time, the ratings for pleasantness and burdensomeness were only slightly related to each other, indicating that the participants interpreted these attributes differently and/or that these attributes measure different aspects of the touch experience.

On the individual level, the picture was less clear. All types of tactile attribute ratings produced variable and inconsistent shapes, and the ratings of the majority of participants followed neither a quadratic nor a linear pattern. In particular, there was high variability for ratings of hard/softness. This indicates that participants were not well able to relate the touch to this attribute. Ratings for the attributes weak/intense and exciting/not exciting were more consistent between individuals than for unpleasant/pleasant, at least for the middle velocities. This is in line with a recent study that demonstrated a large interindividual variability for ratings of touch pleasantness (Croy et al., 2020), and the same was found for odor pleasantness (Sailer et al., 2016). High variability between participants may mask relationships on a group level. It is possible that there are subgroups of participants who are consistent in their ratings, as was the case for raters of odor pleasantness (Sailer et al., 2016). Future studies may be able to identify how and why these subgroups differ. In the following, we will only interpret the group-level data.

### Emotional Attributes

We hypothesized that only positive emotional attributes would follow a u-shaped pattern, whereas sensory attributes would not. When high values represent positive emotions, the curve should be u-shaped, and when high values represent negative emotions, the curve should be inverted u-shaped. The first part of this hypothesis was partly confirmed. The emotional attribute of “burdensome–not burdensome” and “unpleasant–pleasant” followed an inverted u-shape, but “exciting–not exciting” did not.

Whereas “not burdensome” is an emotional attribute, it is not an explicitly positive one. “Not burdensome” rather describes the absence of a negative property. It seems as if participants interpreted the absence of a negative property (burden) in the same way as the presence of a positive emotional property. This would correspond to the Epicurean concept of pleasure as the absence of pain (e.g., Rist, 1972). In line with this, relief occurring in the absence of an expected negative stimulation can be conceptualized as a positive emotion (e.g., Deutsch et al., 2015). In addition, participants appeared to have found the description “not burdensome” even more appropriate for CT-targeted touch than “pleasant,” given that the mean ratings for “not burdensome” were higher than for “pleasant.” This indicates that pleasantness is more than just the absence of “burdensomeness.” These findings also relate to findings from a study where the TPT (Guest et al., 2011) was translated into Swedish (Ackerley, Saar, et al., 2014). Here, a third emotional factor “negative affect” was identified, which is independent from the two other factors “positive affect” and “arousal.” Applied to our results, it could mean that “burdensome–not burdensome” relates to the factor “negative affect,” and “unpleasant–pleasant” to the factor “positive affect.”

### Sensory Attributes

The second part of our hypothesis stating that sensory attributes would not follow an inverted u-shaped pattern was rejected. Ratings for “rough” followed the same pattern, with roughness being rated highest for medium CT optimal velocity, and ratings for smoothness being highest for very fast and very slow brushing. Although no recordings from CT fibers were made in this study, this suggests that CTs may not be specific for the perception of positive emotional touch. Thus, studies are needed that record from CTs while brushing at different velocities and collect ratings on attributes other than pleasantness.

The finding that smooth–rough ratings also follow a quadratic function is somewhat surprising. Whereas CT fibers are believed to convey the affective meaning of touch, roughness/smoothness is typically considered a sensory rather than an emotional attribute (e.g., Guest et al., 2011). However, when investigating labels that were rated differently for touch on CT-innervated versus noninnervated skin, certain sensory attributes (namely, “fluffy” and “hairy”) succeeded at least similarly well, if not even better, than “pleasant” did (McGlone et al., 2012). Smoothness ratings were also different for self-touch and other touch on the palm (Gentsch et al., 2015). Whether smoothness perception differed for the palm and forearm in this study is unfortunately not reported. This means either that CTs may also convey descriptive properties of touch or that the smooth–rough dimension can also be understood to be an affective attribute. Unfortunately, the number of participants in the present study is not large enough to follow up this question with a factor analysis.

In the present study, CT-targeted touch was perceived as rougher than very slow or very fast touch. This is in contrast to studies on fabric perception where fabrics rated as smooth are typically also experienced as pleasant (Ekman et al., 1965; Essick et al., 2010; Etzi et al., 2014; Major, 1895; Ripin & Lazarsfeld, 1937; Verrillo et al., 1999). We can only speculate on why our participants rated CT-targeted touch as roughest, or least smooth. Potentially, the attribute “smooth” has a different connotation in German as it also signifies “slippery.” According to the Leipzig Corpora Collection (Goldhahn et al., 2012), a corpus-based monolingual full-form online dictionary, the second most frequent word co-occurring with “smooth” (glatt) is “street,” indicating that “smooth” is very often used in the sense of “slippery,” which bodes ill in this context. Thus, “smooth” is not always positive.

### Potential Influences of Response Format and Wording of Attributes

Related to this, the valence of attributes other than “smooth–rough” may not be always straightforward. The attribute “exciting” in German can have both positive and negative connotations as it can signify both positive arousal and annoyance. It is also not clear which of the anchors “weak” and “intense” in relation to the received touch would describe a positive experience.

Furthermore, we cannot exclude that participants were confused by the way the rating scales were anchored. For some attributes, the right pole of the scale (coded with +10) described a positive aspect, in other items a negative aspect. This was done to avoid a response set where participants routinely cross all the scales for all velocities at about the same point. Nevertheless, participants may not always have managed to adapt to this change in valence.

Apart from the response format, there are characteristics in the wording of the attributes that may have affected the response. Previous studies identifying characteristics of texture and touch differed in their approach of using bipolar opposites or monopolar factors (Okamoto et al., 2013). This may explain some of the differences in their findings. In the present study, some of the items were bipolar opposites, such as “weak–intense,” and others were monopolar, such as “burdensome–not burdensome.” It is possible that bipolar opposites are perceived as semantically “further apart” from each other than monopolar opposites. For example, “not burdensome” may be interpreted as neutral. If this is the case, then the ratings for monopolar items would be spread out more across the length of the scale than for bipolar opposite items.

It has long been discussed whether emotional states are monopolar or whether each affect has a bipolar opposite (e.g., Lorr et al., 1982; Nowlis & Nowlis, 1956; Russell, 1979). The general consensus now appears to be that emotional states are bipolar. It has also been argued that bipolar opposites are more readily accessible and produce more valid answers (Keren, 2011). According to this view, bipolar opposites trigger associations that are congruent with the opposite. Negations, on the other hand, trigger associations of the negated characteristic, which then are denied and may be incongruent with the intended meaning. This can give rise to memory errors, such that “not warm” was remembered as “not cold” (Mayo et al., 2004). Processing negations is, in general, more complex than processing affirmations (Carpenter & Just, 1975; Clark & Chase, 1972; Mayo et al., 2004; Trabasso et al., 1971; Wason, 1972). We can only speculate how mixing bipolar and monopolar opposites might have influenced the present results. As bipolar and monopolar opposites were used for both emotional and sensory attributes, systematic effects are unlikely. However, the above literature suggests that bipolar opposites are to be preferred.

### Situational Influences on Touch Attributes

In general, studies on attributes to describe the properties of materials differ in the dimensions they identify. Whereas most studies find that emotional attributes load on the factors of positive affect and arousal, one study found a third factor “negative affect” (Ackerley, Saar, et al., 2014), and another study a third factor “dominance,” defined as feelings of control or activity versus feelings of being controlled and passive (Drewing et al., 2018). In the latter study, 47 solid, fluid, and granular materials were rated. In this context, six sensory dimensions emerged, namely fluidity, roughness, deformability, fibriousness, heaviness, and granularity. This revealed some previously not established dimensions, which is presumably due to the large range of materials employed. It also shows that the range of attributes depends on the stimulus material at hand. For the evaluation of different fabrics, for example, rather different attribute pairs such as “crisp–supple,” “bulky–sleazy,” and “thin–lofty” can be applied as well (Brand, 1964). Moreover, ratings of stimulus materials are also intertwined with their intended purpose, for example, when evaluations for silk and rayon fabric were compared (Ripin & Lazarsfeld, 1937).

Further situational factors that have been shown to influence touch attributes are visual influences. For example, CT-targeted stroking was experienced as more pleasant when the own arm was visible (Keizer et al., 2019), and ratings of fabrics were revised after having seen them (Ripin & Lazarsfeld, 1937). Not only visual input but also auditory (Guest et al., 2002) and olfactory (Croy et al., 2014) input can change the evaluation of touch. In addition, actively exploring a material versus being touched passively also influences ratings of pleasantness (Etzi et al., 2014). Furthermore, factors within the participants such as expectations or attitudes (e.g., McCabe et al., 2008), previous experiences (Drewing et al., 2018; Sailer & Ackerley, 2019), the perceived quality of the relation to the touch provider (Triscoli et al., 2017), and possibly personality can influence how touch and materials are rated.

It is clear from all this work that the many different attributes in touch are complex and can be affected by the experimental design, which may link to the varied perception of tactile attributes.

### Limitations

This study lacks a control condition. The inclusion of a nonhairy/non-CT site such as the palm would have lent more substance to the CT specificity of the findings. If those attributes that best differentiated CT optimal touch from other touch would receive different ratings at non-CT sites, this would strengthen the argument that these properties are conveyed by CT afferents, but not by other types of afferents.

Furthermore, the participants did not wear headphones and the sound of the Rotary Tactile Stimulator is slightly different across velocities. It can therefore not be excluded that (some of) the ratings were influenced by the sound.

Other limitations concern the wording of the attributes. The distinction between sensory and emotional attributes is not always clear-cut, as sensory attributes can also have an emotional connotation, such as “warm.” “Warm” can also be used metaphorically to mean emotional warmth, as in a person (e.g., Asch, 1946; Kelley, 1950; Nisbett & Wilson, 1977). Indeed, there appears to be an automatic association between terms describing temperature (warm and cold) and positive and negative valence (Bergman et al., 2015).

As a further limitation, the English words identified as belonging to a sensory or emotional category were simply translated to German, where the meanings may not always be identical. For questionnaires, a cultural adaptation in several steps including back-translation is recommended to ensure understandability, interpretation, and cultural relevance of the translation (e.g., Wild et al., 2005). It appears meaningful to do this also for VAS adjectives that are validated in a different language only.

### What Does This Mean for the Interpretation of CT Activation?

Whereas CT firing was not measured in the current experiment, previous studies linking CT activity to pleasantness ratings were also based on different sessions and different samples. Thus, the correlation between CT firing frequency and pleasantness ratings was not a very strong link. Our findings showed that touch performed at different speeds is evaluated with ratings that follow an inverted u-shape for pleasantness, burdensomeness, and roughness. Thus, ratings for burdensomeness and roughness also show the same shape as CT impulse rate typically does. This indicates that the perception of CT-targeted stimulation can be well described in terms other than pleasantness, including the nonemotional attribute “smooth–rough.” In line with this, recent findings indicate that the neuroanatomical distinction between discriminative and affective touch is not as clear-cut as previously postulated (Marshall et al., 2019). The similar shapes for pleasantness, burdensomeness, and roughness ratings also imply that the “social touch hypothesis” may be too narrow. The “social touch hypothesis” (Morrison et al., 2010; Olausson et al., 2010) states that the role of the CT system is to specifically capture the affective (pleasant) aspects of touch relevant in social interaction. The increased roughness ratings at 3 cm/s stroking found in the present study could imply that CTs also encode texture. Thus, the question of what information CTs actually transmit merits further investigation.

On the other hand, it needs to be kept in mind that percepts of pleasantness, roughness, etc., are generated on a central level. Also, even if we asked for ratings on emotional versus sensory dimensions, what these questions tap into is a cognitive evaluation of the sensation. Thus, in addition to the bottom-up activation from CTs and myelinated afferents, giving the ratings requires a variety of higher processing stages. Participants need to consciously interpret the sensation and the semantics of the evaluation scale before they assess to what extent a particular attribute fits to the sensation. Therefore, it is unknown how much of the eventual rating is due to the input from CT afferents, from myelinated afferents, or to cognitive processes, and how the proportional involvement of these processes differs for the seven attributes used.

It is, for example, possible that CT activation is centrally interpreted as representing those aspects which are most salient for a given stimulation. For stimulation by a brush, these may be pleasantness, burdensomeness, and roughness. For skin-to-skin stimulation on the inner thigh, eroticism may be the most salient dimension. Further studies are needed to investigate this question, ideally with CT recording and ratings on different attributes in the same participants.

### Further Implications

The present findings also have implications for clinical evidence in the field of affective touch where certain groups of participants or patients do not show the typical velocity-dependent pleasantness curve. This was the case for patients with anorexia nervosa (Crucianelli et al., 2016; Davidovic et al., 2018), patients undergoing psychotherapy (Croy, Geide, et al., 2016), and healthy participants who were touch-deprived (Sailer & Ackerley, 2019), among others. Our findings also raise the possibility that these participant groups might not have difficulties in experiencing the pleasantness of CT optimal touch but instead associate the CT touch with different word labels. Taking this further, this could mean that they do not have a disordered perception of affective touch, but mainly a different way to describe it. Whereas this is purely speculative, it would nevertheless be an interesting question to explore in future studies.

## Conclusion

Stroking with CT-targeted velocity is not exclusively linked to experienced pleasantness but can also be perceived as “not burdensome” and “rough.” This leads to the hypothesis that CTs transport a sensation that can be described by a wider range of emotional words than “pleasant,” and that sensation is possibly not even limited to emotional descriptors. Further studies are needed to elucidate the role of the experimental design, context, and individual state for these perceptions.

## Notes

**Publication Ethics:** The study was approved by the Ethical Committee of the Technical University of Dresden. Participation was remunerated with 20 €.

**Open Data:** Data will be made available on request.
